# Vascular Assessment Stratifying Preeclampsia Risk in Overweight/Obese Women

**DOI:** 10.1161/HYPERTENSIONAHA.124.24394

**Published:** 2025-06-18

**Authors:** Christos Chatzakis, Laura A. Magee, Renata Castello, Gerardo Miranda, Peter von Dadelszen, Kypros H. Nicolaides, Marietta Charakida

**Affiliations:** 1 Fetal Medicine Research Institute, King’s College Hospital, London, United Kingdom (C.C., R.C., G.M., K.H.N., M.C.).; 2 Institute of Women and Children’s Health, School of Life Course and Population Sciences, King’s College London, United Kingdom (L.A.M., P.v.D., K.H.N.).; 3 School of Biomedical Engineering and Imaging Sciences, King’s College London, United Kingdom (M.C.).

**Keywords:** body mass index, obesity, preeclampsia, pulse wave analysis, uterine artery

## Abstract

**BACKGROUND:**

Overweight and obesity greatly increase the risk of preeclampsia. There is a need to better risk-stratify these women in pregnancy and channel resources to those who can benefit most.

**METHODS:**

Prospective observational study of 11 962 women with singleton pregnancies attending a routine assessment at 35+0 to 36+6 weeks’ gestation at King’s College Hospital, London, United Kingdom. Women were categorized by their body mass index at 11 to 13 weeks’ gestation as normal weight (18.5–24.9 kg/m^2^), overweight (25.0–29.9 kg/m^2^), or obese (≥30 kg/m^2^). We recorded maternal demographics, assessed uterine artery pulsatility index and ophthalmic artery peak systolic velocity ratio, and measured carotid-to-femoral pulse-wave velocity. Preeclampsia development was retrieved from medical records. Multivariable logistic regression was undertaken to examine determinants of preeclampsia. Mediation analysis was performed to assess causal relationships.

**RESULTS:**

In this cohort, 28.4% were overweight and 17.9% were obese. Preeclampsia developed more often in overweight/obese (versus normal weight) women (6.0% versus 1.7%, respectively; *P*<0.001); women of Black and South Asian ethnicity were at particularly increased risk (*P*=0.02 and 0.004, respectively). Determinants of preeclampsia development did not differ by body mass index. Mediation analysis suggested that the effect of overweight/obesity on preeclampsia development may be mediated partly by changes in maternal cardiovascular indices, particularly aortic stiffness (as reflected by carotid-to-femoral pulse-wave velocity, proportion mediated=72.6%).

**CONCLUSIONS:**

Risk factors for term preeclampsia are largely similar between overweight/obese and normal-weight women, except for Black and South Asian women, who face a particularly high risk within the overweight/obese group. Maternal vascular assessment may serve as a valuable tool for stratifying the risk for term preeclampsia in these populations.

**NOVELTY AND RELEVANCE**

**What Is New?**

This study identifies maternal vascular indices, as significant mediators of the relationship between overweight/obesity and preeclampsia.

Maternal cardiovascular health assessment, is suggested as a potential tool for better risk stratification of term preeclampsia.

**What Is Relevant?**

Hypertension is a core feature of preeclampsia, and this study highlights how vascular changes associated with hypertension may be influenced by maternal body mass index.

The findings point to vascular health assessment as a way to predict hypertensive complications in pregnancy, particularly in high-risk groups.

**Clinical/Pathophysiological Implications?**

Maternal vascular assessment at 35 to 36 weeks could enhance preeclampsia risk stratification.

These findings suggest the potential benefit of interventions targeting vascular health before, during, and between pregnancies to reduce preeclampsia risk and improve overall maternal cardiovascular outcomes.

**See Editorial, pp 1443-1445**

In recent years, the global prevalence of overweight and obesity in women of reproductive age has risen.^1,2^ Approximately 1 in 4 pregnant women is now classified as overweight or obese, based on their prepregnancy body mass index (BMI). This is of great concern to the maternity care community, as elevated BMI (particularly obesity) increases the risk of a broad array of adverse pregnancy outcomes for both mothers and babies.^3–7^ For instance, studies indicate that obese (versus normal weight) women have a 2 to 3× higher risk of developing preeclampsia.^8^

Although overweight and obesity greatly increase the risk of adverse outcomes in pregnancy, it is important to appreciate that the majority of these women, even those with a BMI ≥30 kg/m^2^, have normal, uncomplicated pregnancies. Among 387 British women with a booking BMI >35 kg/m^2^, 75% had an uneventful pregnancy.^9^ Similarly, among >115 000 Canadian women with BMI >30 kg/m^2^, ≈60% had normal pregnancy outcome.^10^ As such, it is important to risk-stratify pregnant overweight or obese women so that resources can be allocated to those at greatest risk of adverse outcomes, who can benefit most from enhanced surveillance and intervention. Our group and others have shown that women at risk of preeclampsia have evidence of hemodynamic and vascular maladaptation in pregnancy, and that assessment of these indices in conjunction with information on maternal characteristics, can better identify women at increased risk of preeclampsia.^11^ In addition, we have recently demonstrated that overweight and obese (versus normal weight) women have altered hemodynamics in pregnancy.^12^ However, it remains unknown whether assessment of these vascular indices among overweight/obese women can better identify those who will develop preeclampsia.^13^

The aim of the current study was to: (1) compare uteroplacental, and maternal hemodynamic and vascular indices at 35 to 36 weeks’ gestation, according to early pregnancy BMI and weight gain between early and late pregnancy; (2) evaluate whether these indices differ in women who develop preeclampsia (versus those who do not); and (3) explore whether these indices make an independent contribution to the development of preeclampsia.

## Methods

### Data Availability

The data that support the findings of this study are available from the corresponding author upon reasonable request.

### Study Design and Participants

This was a prospective observational study of women attending a routine hospital visit at 35+0 to 36+6 weeks’ gestation at King’s College Hospital, London, United Kingdom, between December 2021 and June 2024. This visit included: (1) recording of maternal demographics and medical history; (2) ultrasound examination for fetal anatomy and growth; (3) Doppler studies of the uterine and ophthalmic arteries; and (4) carotid-to-femoral pulse-wave velocity (cfPWV) measurements. Gestational age was determined by measurement of fetal crown-rump length at 11 to 13 weeks’ gestation or fetal head circumference at 19 to 24 weeks.^14^ Women gave written informed consent to participate in the Advanced Cardiovascular Assessment in Pregnancy study (reference 18/NI/0013; Integrated Research Application System ID 237936), which was approved by the NHS Research Ethics Committee. Details of maternal characteristics and the findings of the assessment at 11 to 13 weeks were recorded in our electronic database (Viewpoint).

The inclusion criteria for the study were singleton pregnancies delivering a nonmalformed liveborn neonate. Pregnancies with maternal underweight BMI (<18.5 kg/m^2^), fetal aneuploidies or major abnormalities, and those with chronic hypertension, type 1 or 2 diabetes, systemic lupus erythematosus, or antiphospholipid syndrome were excluded. Women were categorized based on their BMI at antenatal care booking at 11 to 13 weeks’ gestation, as normal weight (BMI 18.5–24.9 kg/m^2^), overweight (BMI 25.0–29.9 kg/m^2^), or obese (BMI ≥30 kg/m^2^).

### Doppler Assessment of the Uterine Arteries

Measurement of mean uterine artery pulsatility index was performed by color flow imaging of the left and right uterine arteries, by transabdominal ultrasound.^15^

### Hemodynamic and Vascular Measurements

#### Ophthalmic Arteries

Assessment of the ophthalmic arteries was performed using a 7.5-MHz linear transducer (Canon Aplio i900 PLT-704SBT Linear Probe, Canon Medical Systems Europe BV, Zoetermeer, the Netherlands). Flow velocity waveforms from the left and right ophthalmic arteries were obtained twice from each eye, and the average was calculated of the 4 measurements of the peak systolic velocity (PSV) ratio.^16^

#### Carotid-to-Fermoral Pulse-Wave Velocity

Aortic stiffness was assessed directly by measuring cfPWV, obtained using the Vicorder device (Skidmore Medical, Ltd, Bristol, United Kingdom), which measures the time taken for the arterial pulse wave to travel from the carotid to the femoral artery. Participants were studied in the supine position, after ≈5 minutes’ rest. The device measures simultaneous pressure waveforms by a volume displacement technique, using blood pressure (BP) cuffs placed around the neck (to pick up the carotid pulse wave) and the right upper thigh (to measure the femoral pulse wave in real time, over at least 10 heartbeats). Both cuffs are automatically inflated and the corresponding oscillometric signal is analyzed to accurately measure, in real time, the pulse time delay and the consequent cfPWV. To calculate transit time, the Vicorder software automatically marks the pulse wave’s steepest ascending part (maximum systolic upstroke) and uses a defined timeframe to detect the wave’s nadir. The shift in time between the marked areas on the carotid and femoral pulse waves, which is the transit time, is detected by cross-correlation. The distance from the carotid-to-femoral pressure cuffs was measured using a tape. To account for differences in abdominal circumference due to the pregnant uterus, and to reduce variability and error in distance assessment, all measurements were performed from the suprasternal notch to the right shoulder and from there to the midpoint of the BP cuff on the thigh. cfPWV was expressed in m/s.^11,17^

#### Pulse Wave Analysis

The waveform of the brachial artery pulse was obtained oscillometrically and analyzed using the Vicorder device. By applying a brachial-to-aortic transfer function, the aortic waveform was generated. Central systolic and diastolic BP, cardiac output, and total peripheral resistance were derived from the pulse wave analysis software. Augmentation pressure was also obtained, and the augmentation index, an indirect measure of arterial stiffness, was expressed as a percentage of central pulse pressure.^11^

### Pregnancy Outcome

Preeclampsia was defined according to the American College of Obstetricians and Gynecologists, as chronic or gestational hypertension with new-onset, from 20 weeks’ gestation, of either proteinuria or other maternal end-organ involvement.^18^ Proteinuria was defined as ≥300 mg in 24 hours or a urinary creatinine ratio ≥30 mg/mmol (0.3 mg/mg) or 2 readings of at least ++ on dipstick analysis of midstream or catheter urine specimens if no 24-hour collection is available. Maternal end-organ involvement was >1 of: pulmonary edema, platelet count <100×10^9^/L, serum creatinine >97μmol/L (1.10 mg/dL) or a doubling in its value, or abnormal liver enzymes (alanine or aspartate aminotransferase >67 IU/L).

### Statistical Analysis

Continuous variables were presented as mean and SD if their distribution was normal, or as medians and interquartile range if the distribution were nonnormal. The distribution was tested using the Kolmogorov-Smirnov test. Categorical variables were summarized as percentages.

ANOVA or Kruskal-Wallis and Bonferroni tests for post hoc analysis were used for between-group comparisons of continuous variables. χ^2^ or Fisher exact tests were used for between-group comparisons of categorical variables.

Multivariable logistic regression was used to assess the effect on preeclampsia development of both BMI (in kg/m^2^) at 11 to 13 weeks’ gestation and weight gain between 11 to 13 and 35 to 36 weeks’ gestation, independent of baseline maternal and pregnancy characteristics. Maternal demographics included in the regression were: maternal age (years), ethnicity (White, Black, South Asian, East Asian, >1), decile of the Index of Multiple Deprivation (IMD) across 7 domains [https://www.gov.uk/government/statistics/english-indices-of-deprivation-2019], complications in previous pregnancies (preeclampsia, gestational diabetes, small-for-gestational age, or large-for-gestational age infant), and family history of preeclampsia or diabetes in a first- or second-degree relative. Pregnancy characteristics included in the regression were: conception by ovulation induction or in vitro fertilization, smoking, and nulliparity. Correction for body surface area was not undertaken for cardiac output and total peripheral resistance measurements, as height and weight were already considered in the multivariable regression. Interaction terms were used to assess whether hemodynamic, vascular indices, and uterine artery Pulsatility Index (PI) effects (if any) on preeclampsia development differed by BMI, comparing overweight/obese women with normal-weight women.

Mediation analysis was conducted using a causal mediation approach, employing the mediate function from the mediation R package. This analysis evaluates the indirect effect of the independent variable (ie, early pregnancy BMI) on the outcome of interest (ie, birth with preeclampsia), through a mediator (ie, uterine artery PI, and hemodynamic and vascular measurements), known as the Average Causal Mediation Effect. This analysis represented the sum of the direct effect (average direct effects) of early pregnancy BMI on birth with preeclampsia, bypassing the mediators, as well as the indirect effect. The Average Causal Mediation Effect, direct, and indirect effects, as well as the proportion of effect mediated, were tested for statistical significance, using nonparametric bootstrap methods, creating CIs by repeatedly resampling the data to provide robust estimates, even in complex models.

The statistical software package R 2.15.1 (The R Foundation for Statistical Computing) was used for data analysis.^19^

## Results

### Participant Characteristics

Of the 11 731 women in our study population, 3403 (29.0%) were overweight, 2136 (18.2%) were obese, and 6192 (52.8%) were of normal weight. Preeclampsia developed more often among overweight/obese women (n=204/5539, 3.7%; *P*<0.001) than among normal-weight women (n=141/6192, 2.3%). There were only 10 cases of preeclampsia that developed before 37 weeks’ gestation.

Table 1 provides a direct comparison of early pregnancy characteristics according to BMI and subsequent development of preeclampsia. Among overweight/obese and normal-weight women, those who developed preeclampsia (versus those who did not) differed according to many baseline characteristics. Among women who developed preeclampsia (gray columns, Table 1), overweight/obese (versus normal weight) women were: younger; more likely to be of Black or South Asian ethnicity, and more likely to have a family history of diabetes. Among women who did not develop preeclampsia, overweight/obese (versus normal weight): had lower gestational weight gain; were more likely to be of Black and less likely to be of East Asian ethnicity; were less likely to conceive following in vitro fertilization; were more likely to smoke, have a family history of diabetes, be nulliparous, and have had prior preeclampsia, GDM, or a large-for-gestational age infant; and had an IMD that reflected social deprivation.

**Table 1.**
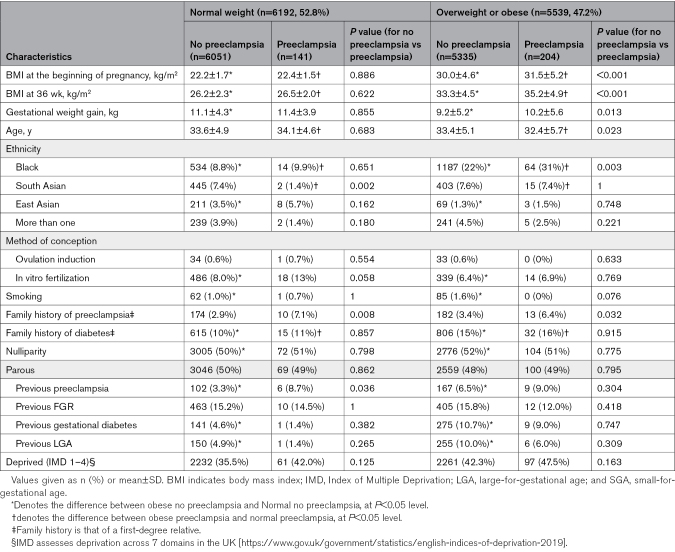
Maternal Characteristics by Early Pregnancy BMI, and Birth With Preeclampsia

### Maternal Hemodynamic and Vascular Indices

Table 2 shows that the gestational age at assessment was similar for overweight/obese and normal-weight women, and according to development of preeclampsia (or not). Among both overweight/obese and normal-weight women, most indices examined had higher values among those who developed preeclampsia, compared with those who did not (Table 2). Among overweight/obese women, those who developed preeclampsia (versus those who did not), had significantly different values for most indices assessed: higher uterine artery PI, mean arterial pressure, central systolic and diastolic BP, cardiac output, and cfPWV, and lower ophthalmic artery PSV ratio. Cardiac output adjusted for BMI, augmentation index, and total peripheral resistance did not differ between groups. Among normal-weight women, those who developed preeclampsia (versus those who did not) also had significantly different values for most indices assessed: higher uterine artery PI, mean arterial pressure, central systolic and diastolic BP, cfPWV, total peripheral resistance, and ophthalmic artery PSV ratio. Augmentation index, cardiac output, and cardiac output adjusted for BMI did not differ between groups.

**Table 2.**
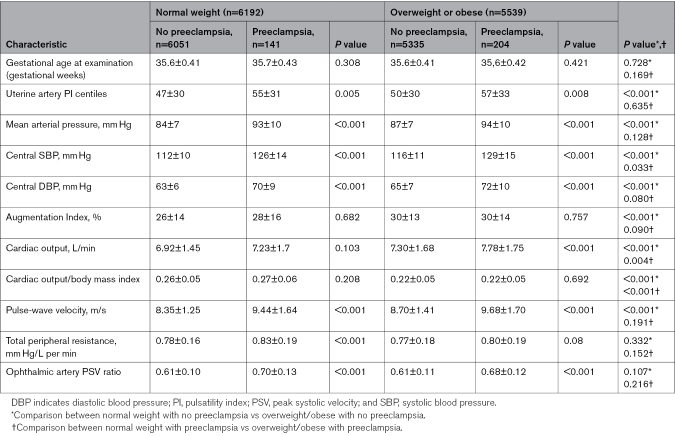
Hemodynamic and Vascular Indices by Obesity and Preeclampsia Status

### Determinants of Preeclampsia Development According to BMI Category (Multivariable Analysis)

Table 3 reports determinants of preeclampsia development, independent of early pregnancy BMI and gestational weight gain (between the first and third trimesters). From early pregnancy characteristics, younger maternal age, family history of preeclampsia, Black or South Asian ethnicity, and more deprivation (by IMD) were independent determinants of preeclampsia development (as indicated by yellow shading, Table 3). From hemodynamic assessment and vascular indices, determinants of preeclampsia development were: higher uterine artery PI, cfPWV, and ophthalmic artery PSV ratio. Interaction analysis revealed that women of Black or South Asian ethnicity had a particularly high risk of preeclampsia development in association with being overweight/obese.

**Table 3.**
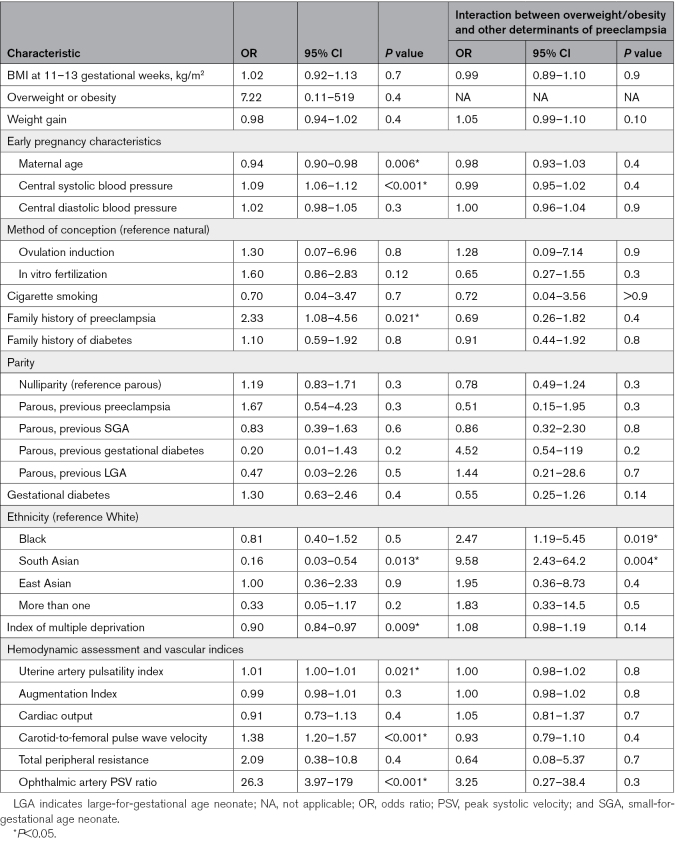
Determinants of Preeclampsia Development in All Women*

### Mediation Analysis

From the hemodynamic and vascular indices assessed, ophthalmic artery PSV and cfPWV and were found to be mediators of the BMI and preeclampsia relationship (Average Causal Mediation Effect *P* values of 0.024 and <0.001, respectively). Furthermore, a large proportion of the effect of BMI on development of preeclampsia was explained through cfPWV (proportion mediated of 73.0%); although the *P* value for the proportion mediated was not statistically significant (*P*=0.14). A smaller proportion of the effect of BMI on development of preeclampsia was explained by ophthalmic artery PSV (29.7%, *P*=0.608). Neither BMI itself nor gestational weight gain was found to directly affect the development of preeclampsia.

## Discussion

### Main Findings

Almost half of this cohort of >11 000 women were overweight or obese in early pregnancy. The incidence of preeclampsia was higher among overweight/obese (versus normal weight) women, and Black and South Asian ethnicity conferred higher risk for preeclampsia development in overweight/obese women. Gestational weight gain, early pregnancy characteristics, and 35- to 36-week measurement of uterine artery PI, and hemodynamic and vascular indices, differed according to baseline BMI and subsequent development of preeclampsia. However, in multivariable logistic regression, neither BMI (assessed as a continuous variable or categorically) nor gestational weight gain were associated with preeclampsia. Independent risk factors for preeclampsia were elements of maternal characteristics (ie, maternal age, central systolic BP, family history of preeclampsia, South Asian ethnicity, and lower IMD) and cardiovascular assessment (ie, uterine artery PI, cfPWV, and ophthalmic artery PSV ratio). Mediation analysis suggested that the effect of overweight/obesity on preeclampsia development may be mediated partly by changes in maternal cardiovascular indices, particularly aortic stiffness (as reflected by cfPWV), but also potentially to a lesser extent, by changes in the peripheral circulation (as reflected by ophthalmic artery PSV ratio).

### Interpretation of Results and Comparison of Findings From Previous Studies

Our findings suggest that risk factors for preeclampsia development are largely similar in overweight/obese and normal weight women, and that assessment of aortic stiffness may be helpful to risk-stratify overweight/obese women for preeclampsia development, independent of maternal history and uterine artery Doppler.

In the current study, consistent with previous observations, we saw that the incidence of preeclampsia was higher in overweight/obese (versus normal weight) women^3,8,9^ and among women of Black or South Asian ethnicity.^20^ However, to our knowledge, it has not been previously demonstrated that women of Black and South Asian ethnicity are at particularly high risk to develop preeclampsia when they are overweight/obese.

A recent systematic review has reported specific predictors of adverse maternal and newborn outcomes among women with obesity, to better risk-stratify this population.^7^ These predictors included non-White ethnicity and maternal age (<20 years and ≥35 years), as in our study, along with abdominal obesity, prior bariatric surgery, and prepregnancy type 1 diabetes^7^; however, cardiovascular indices were not assessed. Other predictors of preeclampsia identified in our study are consistent with previous reports, irrespective of maternal adiposity status: maternal age, family history of preeclampsia, IMD, and higher uterine artery PI, cfPWV, and ophthalmic artery PSV ratio.^11,16,21,22^ In a recent Canadian study in 87 singleton pregnancies, overweight/obese pregnant women had increased aortic stiffness, and among those who developed preeclampsia, more unfavorable vascular responses.^23^

The pathophysiology of preeclampsia in obese women is not fully understood, and various mechanisms have been suggested.^24^ Adipose tissue is abundant in inflammatory mediators and complement proteins, which may contribute to altered placental angiogenesis and development of preeclampsia.^25^ In addition, maternal obesity is characterized by decreased angiogenic regulators and an increase in oxidative stress markers.^25,26^ This environment may impact endothelial integrity and lower the threshold for development of endothelial dysfunction and arterial stiffness. Considering that central arteries (ie, the aorta) might be more vulnerable to atherosclerosis, and increased cfPWV can predict adverse outcomes such as cardiovascular events and mortality in adults,^27^ our data further support the link between obesity, preeclampsia, and future increased cardiovascular risk.

In our study, in overweight/obese women, cfPWV was increased, but augmentation index was not. Although the 2 measures are used to assess arterial stiffness, they do not always behave in parallel. Stiffer arteries lead to higher PWV, whereas, augmentation index reflects the contribution of the reflected wave to central BP. Thus, the latter depends on both arterial stiffness and the timing of wave reflection. In particular, in obese individuals, the larger body size and increased distance between the heart and peripheral reflection sites can alter the timing of reflected waves. This may reduce the augmentation index, even when aortic stiffness is increased.^28^

There are effective interventions to improve maternal vascular health outside pregnancy. Although maternal age and family history of preeclampsia cannot be modified in a woman’s life course, and IMD can be addressed only outside the health care realm, weight reduction can modify cardiovascular and metabolic risk factors to improve vascular health and prognosis in individuals with overweight or obesity, and so this is a potential prepregnancy intervention.^29–32^ For example, Nordstrand et al^33^ showed a clinically important mean decline in aortic stiffness of 0.6 m/s (95% CI, 0.4–0.8 m/s) following a combined intensive lifestyle intervention of a 1000‐kcal/d calorie-restricted diet and supervised moderate-to-high-intensity exercise training. Similarly, in a meta‐analysis of 1659 individuals (20 studies), weight loss of 8% resulted in a favorable decrease in cfPWV of 0.32 m/s (95% CI, 0.24–0.41 m/s; *I*^2^=26%).^34^ There is also the potential that pravastatin may improve vascular health in pregnancy and reduce preeclampsia.^35^

Hemodynamic assessment in our study revealed that cardiac output was increased in overweight/obese (versus normal weight) women, and in association with development of preeclampsia; however, total peripheral resistance was higher but not statistically significantly increased. While increased cardiac output and increased total peripheral resistance have been reported to be increased in women who developed preeclampsia,^36,37^ and neither of these hemodynamic parameters showed independent association with preeclampsia in our data, this suggests that pharmacological manipulation of cardiac output or peripheral vascular resistance may not be useful in overweight/obese women to reduce the incidence of preeclampsia.

Finally, apart from higher BMI, greater gestational weight gain has been associated with an increased risk of preeclampsia, leading guidelines to advise minimal weight gain in this group of women.^4,9,38^ While overweight/obese (versus normal weight) women in our study did gain less weight from the first to third trimesters of pregnancy, more limited weight gain did not contribute favourably to preeclampsia development once other risk factors and vascular indices were accounted for.

### Strengths and Limitations

Our study has many strengths. We studied a large population of women undergoing routine pregnancy care, rather than small selected groups, to assess the role of both elevated BMI and vascular health on development of preeclampsia. We measured aortic stiffness directly using cfPWV, as the technique has been validated for use in pregnancy and has shown promise in the prediction of preeclampsia from as early as 11 to 13 weeks’ gestation.^39^ By performing mediation analysis, we explored mechanisms of preeclampsia development, and revealed that the effect of BMI on preeclampsia development appears to be mediated, at least in part, by vascular changes.

Our study has limitations. Our estimate of adiposity was BMI, but we recognize that rather than the quantity of fat mass, the pattern of fat distribution (such as central rather than gluteofemoral), about which we lacked information, might also be important in determining preeclampsia development, as it is for future cardiovascular risk.^40–42^ Our hemodynamic and vascular assessments were undertaken in the third trimester of pregnancy, and so we cannot confirm that they did not represent subclinical preeclampsia, or that they persisted postpartum. We lacked information about other cardiovascular risk factors, such as dyslipidemia and insulin resistance, and it is possible that abnormalities in these parameters may also contribute to development of preeclampsia. Our study was performed at 35 to 36 weeks’ gestation, and we are therefore unable to determine whether our findings are applicable to women who develop preterm preeclampsia.

### Clinical Perspective

Traditional risk assessment for term preeclampsia relies primarily on maternal history and demographic characteristics. This study supports the integration of maternal vascular health measures, such as aortic stiffness (cfPWV), into risk assessment protocols, particularly for overweight and obese women. Identifying vascular dysfunction early may enable more precise monitoring and intervention, reducing adverse outcomes. Moreover, lifestyle modifications and vascular-targeted interventions before or during pregnancy—such as weight loss programs or emerging therapies like pravastatin—could improve vascular health and lower preeclampsia risk. Future work should focus on the long-term cardiovascular implications of these vascular changes and assess the feasibility of incorporating vascular assessments into routine antenatal care.

### Novelty and Relevance

This study contributes novel insights into the relationship between maternal obesity, ethnicity, and preeclampsia risk. While previous research has established that obesity increases the incidence of preeclampsia, this study is the first to highlight that Black and South Asian women face disproportionately higher preeclampsia risk when overweight or obese. It further identifies maternal vascular indices, particularly aortic stiffness (cfPWV) and ophthalmic artery PSV ratio, as mediators of the relationship between overweight/obesity and preeclampsia. Unlike earlier studies that focused predominantly on demographic or historical factors, this research demonstrates that vascular assessments can offer independent predictive value, opening avenues for targeted risk stratification. The findings also suggest potential pathways through which obesity influences vascular health, linking these changes to both preeclampsia development and long-term cardiovascular risk.

### Conclusions

Our study highlights that risk factors for term preeclampsia development are similar among overweight/obese and normal weight women, with the exception of Black and South Asian ethnicity. Both maternal characteristics (ie, demographics and prior history) and vascular indices are key, independent risk factors for preeclampsia. These findings suggest that maternal vascular assessment might be a useful adjunct to risk-stratify pregnant women for preeclampsia development, particularly for overweight/obese women who are at particularly high risk of preeclampsia. If future work shows that vascular abnormalities demonstrated in this study at 35 to 36 weeks’ gestation are sustained postpartum, study would be warranted of interventions to improve vascular health before or between pregnancies, or even during pregnancy.

### Perspectives

This study provides new insights into the role of maternal cardiovascular health in the development of term preeclampsia among overweight and obese women. The findings underscore the potential of maternal vascular assessments as valuable tools for risk stratification during pregnancy.

Future research should explore whether the vascular abnormalities observed in late pregnancy persist postpartum, paving the way for interventions that target maternal vascular health before or between pregnancies. Such approaches could help mitigate not only preeclampsia risk in subsequent pregnancies but also long-term cardiovascular health risks in these populations. In addition, studies are needed to evaluate the clinical utility and cost-effectiveness of integrating vascular assessment into routine prenatal care, as well as interventions that could improve vascular function during pregnancy. Overall, these findings provide a foundation for addressing the significant burden of preeclampsia in overweight and obese women, with implications for personalized maternal care and broader public health strategies.

## Article Information

### Sources of Funding

The study was supported by a grant from the Fetal Medicine Foundation (Charity no: 1037116). This body had no involvement in the study design; in the collection, analysis and interpretation of data; in the writing of the report; and in the decision to submit the article for publication.

### Disclosures

None.
